# A Meta‐Analysis of the Impact of Natural Disasters on Internalizing and Externalizing Problems in Youth

**DOI:** 10.1002/jts.22292

**Published:** 2018-06-05

**Authors:** Sonia L. Rubens, Erika D. Felix, Erin P. Hambrick

**Affiliations:** ^1^ Department of Counseling Psychology Santa Clara University Santa Clara California USA; ^2^ Gevirtz Graduate School of Education University of California, Santa Barbara Santa Barbara California USA; ^3^ Department of Psychology University of Missouri–Kansas City Kansas City Missouri USA

## Abstract

Although exposure to natural disasters can lead to diverse mental health (MH) outcomes in youth, most child disaster MH research has focused on posttraumatic stress symptoms (PTSS). To highlight the likelihood of other MH outcomes, we meta‐analyzed studies that have examined other (non‐PTSS) internalizing and externalizing behavior problems in youth exposed to natural disasters. We used PRISMA guidelines to systematically gather studies that have examined the association between natural disaster exposure and non‐PTSS internalizing and/or externalizing problems in samples of children and adolescents. Analyses of random effects models of 62 studies examining non‐PTSS internalizing problems and 26 studies examining externalizing problems showed exposure to natural disasters was significantly associated with non‐PTSS internalizing, *r*_mean_ = .18, *k* = 70, and externalizing problems, *r*_mean_ = .08; *k* = 31, in youth. Moderator analyses revealed a stronger association between disaster exposure and non‐PTSS internalizing problems in countries with a “medium” Human Development Index (HDI) ranking, *r* = .56, than in countries with “high,” *r* = .15, and “very high,” *r* = .16, HDI rankings. We also found a stronger association between disaster exposure and externalizing problems in countries with a medium HDI ranking, *r* = .54, versus high, *r* = .05, and very high, *r* = .04, HDI rankings, and based on parent, *r* = .16, compared to child, *r* = −.01, report. Results support the need for assessment of multiple postdisaster MH outcomes to inform comprehensive interventions. We also include a discussion of the state of the disaster MH research.

## Resumen

Spanish Abstracts by the Asociación Chilena de Estrés Traumático (ACET)

UN METAANÁLISIS DEL IMPACTO DE LOS DESASTRES NATURALES EN LOS PROBLEMAS DE INTERNALIZACIÓN Y EXTERNALIZACIÓN EN LA JUVENTUD

DESASTRES NATURALES Y SALUD MENTAL EN LA JUVENTUD

Aunque la exposición a desastres naturales puede conducir a diversos resultados de salud mental (SM) en los jóvenes, la mayoría de las investigaciones sobre SM infantil en desastres se centran en los síntomas de estrés postraumático (SEPT). Para destacar la probabilidad de otros resultados de SM, hemos realizado un meta‐análisis sobre los estudios que han examinado otros problemas de comportamiento de internalización y externalización (no SEPT) en jóvenes expuestos a desastres naturales. Utilizamos las pautas de PRISMA para recopilar sistemáticamente los estudios que han examinado la asociación entre la exposición a desastres naturales y los problemas de internalización y externalización no SEPT en muestras de niños y adolescentes. Análisis de modelos de efectos aleatorios de 62 estudios que examinan problemas de internalización no SEPT y 26 estudios que examinan los problemas de externalización mostraron que la exposición a desastres naturales se asoció significativamente con problemas de internalización no SEPT, *r _mean_* = .18 (*k* = 70 tamaños de efecto) y externalización, *r _mean_* = .08 (*k* = 31 tamaños de efecto) en la juventud. Los análisis del moderador revelaron una asociación más fuerte entre la exposición a desastres y problemas de internalización no SEPT en países con un índice de desarrollo humano “mediano” (HDI en su sigla en inglés) ranking, *r* = .56, que en los países con clasificaciones HDI “alto”, *r* = .15, y “muy alto”, *r* = .16. También encontramos una asociación más fuerte entre la exposición al desastre y los problemas de externalización en países con una clasificación HDI media, *r* = .54, versus clasificaciones HDI alta, *r* = .05, y muy alta, *r* = .04, y basado en el informe reporte de los padres *r* = .16, en comparación con el del niño, *r* = ‐.01. Los resultados apoyan la necesidad de la evaluación de múltiples resultados de SM posteriores al desastre para informar las intervenciones integrales. También incluimos una discusión sobre el estado de la investigación de SM en los desastres.

## 抽象

Traditional and Simplified Chinese Abstracts by AsianSTSS

The Impact of Natural Disasters on Internalizing and Externalizing Problems in Youth: A Meta‐Analysis

Traditional Chinese

標題: 天災對青少年的內化和外化問題影響元分析

撮要: 縱使經歷天災可導致青少年有多種心理問題(MH), 大部分對兒童災後的MH研究都只針對創傷後壓力症狀 (PTSS)。為了解其他MH的可能性, 我們搜尋檢視經歷天災的青少年其他(非PTSS)內化和外化行為問題的研究, 進行元分析。我們根據PRISMA的指引, 系統性地收集有關兒童及青少年的非PTSS內化和/或外化問題跟天災經歷的關連研究;運用隨機效應模型, 分析62個檢視非PTSS內化問題和26個檢視外化問題的研究。結果發現, 天災經歷跟青少年的非PTSS內化問題 (r_mean_ = .18; (k = 70 效應量)) 和外化問題 (r_mean_ = .08; (k = 31 效應量)) 都有顯著關連。調節因素分析顯示, 相比人力發展指數(HDI)為「高」(r = .15)與「極高」(r = .16)的國家, 在HDI為「中等」的國家裡, 天災經歷跟非PTSS內化問題的關連較強(r = .56)。同樣地, 相比HDI為「高」(r = .05)與「極高」(r = .04)的國家, 天災經歷跟外化問題的關連在HDI為「中等」的國家亦較強(r = .54);根據家長(r = .16)和孩子(r = ‐.01)的報告亦有同樣發現。結果反映, 我們有需要對多種災後的MH進行評估, 以提供更全面的干預治療。我們亦討論到災後MH研究目前的發展。

标题: 天灾对青少年的内化和外化问题影响元分析

撮要: 纵使经历天灾可导致青少年有多种心理问题(MH), 大部分对儿童灾后的MH研究都只针对创伤后压力症状 (PTSS)。为了解其他MH的可能性, 我们搜寻检视经历天灾的青少年其他(非PTSS)内化和外化行为问题的研究, 进行元分析。我们根据PRISMA的指引, 系统性地收集有关儿童及青少年的非PTSS内化和/或外化问题跟天灾经历的关连研究;运用随机效应模型, 分析62个检视非PTSS内化问题和26个检视外化问题的研究。结果发现, 天灾经历跟青少年的非PTSS内化问题 (r_mean_ = .18; (k = 70 效应量)) 和外化问题 (r_mean_ = .08; (k = 31 效应量)) 都有显著关连。调节因素分析显示, 相比人力发展指数(HDI)为「高」(r = .15)与「极高」(r = .16)的国家, 在HDI为「中等」的国家里, 天灾经历跟非PTSS内化问题的关连较强(r = .56)。同样地, 相比HDI为「高」(r = .05)与「极高」(r = .04)的国家, 天灾经历跟外化问题的关连在HDI为「中等」的国家亦较强(r = .54);根据家长(r = .16)和孩子(r = ‐.01)的报告亦有同样发现。结果反映, 我们有需要对多种灾后的MH进行评估, 以提供更全面的干预治疗。我们亦讨论到灾后MH研究目前的发展。

Natural disasters are potentially traumatic events due to their disruptive nature, high extent of impact, production of terror and horror scenes, undesirable and uncontrollable occurrences, and prolonged alterations in the social and material environment. The increasing scientific understanding of the impact of disasters on child and adolescent mental health (MH) has culminated in several reviews that include youth MH concerns (e.g., Bonanno, Brewin, Kaniasty, & La Greca, 2010; Norris, Friedman, Watson, Byrne, & Kaniasty, 2002) and meta‐analyses of postdisaster posttraumatic stress symptoms (PTSS; Furr, Comer, Edmunds, & Kendall, 2010) and depression (Tang, Liu, Liu, Xue, & Zhang 2014) in youth. However, gaps remain in the empirical knowledge of different MH outcomes, as research has overwhelmingly focused on posttraumatic stress symptoms (PTSS). This narrow focus on PTSS has overshadowed the risk that natural disasters pose for other problems.

Youth reactions to disasters include a range of internalizing (e.g., depression and anxiety) and externalizing (e.g., aggression, disruptive behavior, and conduct problems) behavior problems. A better understanding of the likelihood of these diverse reactions has direct implications for screening, diagnosis, and treatment of disaster‐exposed youth. The primary aim of this study was to conduct a meta‐analysis of the literature on non‐PTSS internalizing and externalizing behavior problems among youth exposed to natural disasters. As such, we sought to isolate, to the degree possible, the risk of disaster exposure for non‐PTSS problems. This focus provided needed clarity regarding the degree of association between disaster exposure and other internalizing and externalizing problems as well as important moderators of these outcomes. We examined youth only, as postdisaster reactions have been understudied in this population compared to adults and postdisaster responses of youth may differ from adults given developmental differences at the time of exposure (Dunn, Nishimi, Powers, & Bradley, 2017). Indeed, Norris and colleagues (2002) noted in their review that youth may be more at risk for postdisaster MH problems than adults.

Natural disasters can increase the risk for symptoms of internalizing behavior problems in youth beyond PTSS (e.g., Felix et al., 2011; Lai, LaGreca, Auslander, & Short, 2013). Using structured clinical interviews to assess whether youth met diagnostic criteria for a disorder, Felix et al. (2011) found that PTSD was the least prevalent internalizing disorder in the long‐term aftermath of a disaster, with no difference in PTSD rates in exposed and nonexposed samples. Instead, major depression, social phobia, and separation anxiety were more common, and there were significant differences between exposure groups (Felix et al., 2011). Likewise, in a study of depression and PTSS in youth exposed to Hurricane Ike, Lai et al. (2013) found that at 8 months postdisaster, 13% of exposed youth reported clinical elevations in PTSS and 11% showed elevations in depression. However, at 15 months postdisaster, depression symptoms (11%) were more likely than PTSS (7%; Lai et al., 2013). The results of these studies highlight the need to focus on a range of reactions beyond PTSS.

Ongoing stress, multiple losses, and unyielding reminders of the disaster lead to a sense of helplessness and withdrawal (Bonanno et al., 2010) among exposed individuals, increasing the risk of depression symptoms. In a meta‐analysis of only studies using odds ratio or risk ratio data, Tang et al. (2014) found that natural disaster exposure was associated with depression in youth and adults. Additionally, Goenjian et al. (1995) found that children may also experience fear or intense sadness upon separation from caregivers following a natural disaster. Clearly, there is a range of internalizing problems children may experience after exposure to a natural disaster. Yet, no study of which we are aware has meta‐analyzed the existing literature that examines the impact of natural disasters on a range of non‐PTSS internalizing problems in youth. This is a needed next step to raise awareness of the likelihood and importance of these additional issues.

Children may also exhibit a range of externalizing problems postdisaster, such as disruptive behavior, aggression, and delinquency. Recent research has indicated that externalizing problems are associated with youth disaster exposure, perhaps indirectly through parental distress. Three years post–Hurricane Katrina, mothers who reported significant hurricane‐related distress reported more externalizing problems in their children, such as impulsivity, than mothers with less hurricane‐related distress (Lowe, Godoy, Rhodes, & Carter, 2013). In a longitudinal study, exposure to Hurricane Katrina predicted low maternal mood, which then predicted children's externalizing problems (Scaramella, Sohr‐Preston, Callahan, & Mirabile, 2008).

There have been mixed findings in the literature on postdisaster externalizing problems, which meta‐analysis can help address. Some research has suggested that externalizing problems may not become evident until 1 year or more after a natural disaster (Lowe et al., 2013; Scaramella et al., 2008), whereas other studies did not find an association between disaster exposure and externalizing disorders at approximately 18 months postdisaster (Felix et al., 2011). As more studies examining postdisaster externalizing problems are published, aggregating the information across studies that use various methodologies and measurement time points postdisaster may help estimate how disaster exposure is associated with externalizing problems.

Existing disaster research varies across several important moderating factors. Age may influence postdisaster outcomes in youth given the increase in rates of internalizing and externalizing concerns that occur as children become adolescents (Costello, Mustillo, Erkanli, Keeler, & Angold, 2003). Adolescents, who are already at an increased risk of developing internalizing and externalizing symptoms, may be more likely than young children to develop these symptoms following disaster exposure. Alternatively, young children may be more vulnerable to disaster exposure given the relative influence of early traumatic experiences on developmental trajectories compared to traumatic experiences that occur later in life (Dunn et al., 2017).

Informant discrepancies in child MH also warrant consideration (De Los Reyes & Kazdin, 2005). Although research has suggested that children may be the best reporters of their internalizing behavior problems, their reports may not be reliable until about 8 years of age (Riley, 2004). Reliance on one reporter leads to discrepancies in results across studies and an incomplete picture of the problems exhibited and settings in which they are evident (De Los Reyes & Kazdin, 2005). Thus, a meta‐analytic approach that includes studies of various reporters and accounts for differences in reporters when predicting outcomes is needed.

Disaster location is another key moderating factor. Natural disasters may cause more damage and destruction in the developing world than they do in developed countries, possibly due to poverty and infrastructure issues (Norris et al., 2002). Therefore, it is important to assess for the differential impact of a natural disaster in relation to the available resources in the country in which it occurred. Finally, it is important to consider the timing of when postdisaster psychopathology is assessed. Youth who are highly exposed to a disaster may experience prolonged psychological distress (Felix et al. 2011). For PTSS in youth, most cases occur within 6 months of disaster exposure, with about one‐third of individuals recovering within the first year of onset, and another third continuing to exhibit symptoms many years later (Yule, Bolton, Udwin, O'Ryan, & Nurrish 2000). However, the onset of other internalizing and externalizing problems may not follow a similar pattern. Posttraumatic stress disorder may be a risk factor for the development of some disorders, such as depression (Bonanno et al., 2010). As externalizing problems may arise in an indirect manner following disaster exposure, these problems may appear months or years later rather than directly after the disaster occurs. Examining the timing of assessment of psychological problems will improve the understanding of the link between natural disasters and internalizing and externalizing behavior in youth.

The nature of individual disasters, methods used, outcomes assessed, and populations studied in youth disaster MH research have been diverse. Measures of disaster exposure have varied greatly across studies; this makes it difficult to know whether observed outcomes are due to the degree of exposure alone (McFarlane & Norris, 2006). In addition, there has been an overwhelming focus on PTSS in postdisaster studies dealing with youth (Bonanno et al., 2010), with far fewer studies exploring other problems. The near exclusive focus on PTSS has diminished the attention paid to other possible internalizing and externalizing outcomes among youth and made it difficult to understand how natural disaster exposure is associated with other outcomes. The one meta‐analysis (of which we are aware) that examined non‐PTSD MH problems in youth (Tang et al., 2014) did not address this problem, because the authors focused on a singular diagnosis, included nonnatural disasters, and limited their included studies to those using particular statistics.

In the current study, we meta‐analyzed the existing literature that has addressed the impact of natural disaster exposure on internalizing problems (other than PTSS) and externalizing problems in youth. Aggregating results through meta‐analysis provides greater certainty in observed associations between disaster exposure and internalizing and externalizing problems. Although there are additional emotional and behavioral problems youth may experience beyond these areas, such as substance use, psychosis, and social problems, there have not been enough studies published in these individual areas to date to warrant inclusion in the current meta‐analysis. We focused on natural disasters because person‐made disasters (e.g., technological accidents and mass violence), relative to natural disasters, can have different meaning, impact, and recovery issues, such as community response (McFarlane & Norris, 2006), that warrant separate investigation. We expected to find a positive association between disaster exposure and non‐PTSS internalizing and externalizing problems. We also ran exploratory analyses to examine whether risk for these problems was moderated by age, person reporting psychopathology, time elapsed between disaster and assessment, and resources of the country in which the disaster occurred.

## Method

### Systematic Literature Search

**Figure 1:**
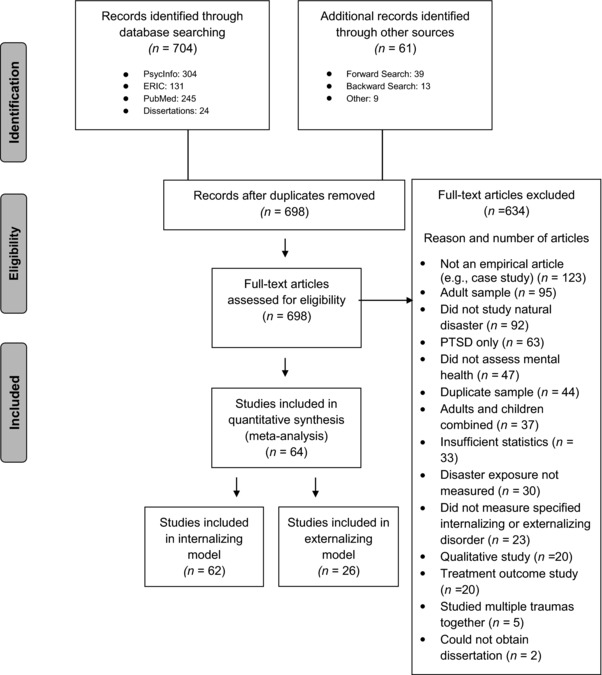
PRISMA flow diagram. There were 24 studies used in both the internalizing and externalizing models. PTSD = posttraumatic stress disorder.

We followed the PRISMA guidelines (Moher, Liberati, Tetzlaff, Altman, & The PRISMA Group, 2009) in conducting our comprehensive literature search (Figure 1). In consultation with a library scientist, we searched PsycINFO and ERIC with Boolean operators using disaster‐related subject heading terms (*disasters* OR *natural disasters)* AND terms related to internalizing and externalizing problems (*disorders* OR *mental disorders* OR *emotional disturbances* OR *adjustment* OR *mental health* OR *behavior disorders* OR *internalization* OR *externalization*). We searched only for English‐language, peer‐reviewed articles and dissertations that included at least one of the following age groups: childhood, neonatal, infancy, preschool age, school age, or adolescence. In PubMED, we searched with medical subject headings with Boolean operators using the terms noted earlier, plus the phrase *psychological adaptation* (which is the most relevant PubMED heading) AND child* OR adolescen* OR teen* OR *youth* OR *minor*. We searched only for peer‐reviewed, English‐language studies of humans. This search was conducted on January 29 and 30, 2016 and updated on July 10, 2017. To ensure search inclusivity, we conducted “forward searching” to find articles that cited studies found in our search. We also conducted “backward searching” by examining studies listed in the reference sections of studies found in our search. We also added articles not found during the search, basing inclusion on our experience with the disaster MH literature and consultations with experts in the field. We sent an email to relevant listservs to obtain unpublished data but received no responses.

### Inclusion Criteria

We employed several a priori inclusion criteria to determine eligibility. “Disaster” was defined, based on the Task Force on Psychological Responses of Children to Natural and Man‐Made Disasters, as a relatively sudden, highly disruptive, time‐limited, and public event (Vogel & Vernberg, 1993). We excluded studies if they focused on person‐made or technological disasters and terrorism. Measures of disaster exposure could include a survey of objective or subjective exposure items, geographic proximity to the disaster, or residence in a shelter versus an unaffected community. Included studies had to be published in English and the primary sample of participants had to have been under 18 years of age. The study had to have examined the impact of disaster exposure on non‐PTSS internalizing and/or externalizing problems; studies that only measured PTSS were excluded. Studies also needed to include a quantitative measure (continuous or categorical) of one of the desired outcomes; qualitative studies were excluded. The studies had to have had an effect size or enough information for us to calculate an effect size. We contacted authors if a relevant study did not provide enough data for us to calculate an effect size; if we received no response, the study was excluded (*n* = 7). When multiple publications used the same sample, the earliest published study was included to avoid nonindependence. We made two exceptions: (a) when the relevant effect size was not available in the first publication but was available in the later publication (Lau et al., 2013), and (b) when a later publication provided an effect size for both internalizing and externalizing problems (Vigil, Geary, Granger, & Flinn, 2010). One study in the internalizing model (Banks & Weems, 2014) used the same sample as another study in the externalizing model (Scott, Lapré, Marsee, & Weems, 2014). Figure 1 lists exclusion data. Appendix A lists all included articles, and Appendix B includes descriptive data on included articles.

### Coding

The study authors completed coding, with regular meetings to ensure consistency. We completed reliability coding on 15% of the articles. Few discrepancies were found (less than 10%) and were resolved through consensus. Effect size data were extracted from each study. We coded studies for sample size, demographics, country in which the study occurred, disaster type and specific disaster studied, and the exposure and outcome measures, to understand the state of the studies included in the analysis. Four study characteristics were coded for moderator analyses: time since disaster, age, Human Development Index (HDI) ranking of the country, and reporter of MH outcomes. Time since disaster was the average length of time between the disaster and the assessment of outcome variables, coded as either less than 1 year or 1 year or more postdisaster. The mean age of the study sample was extracted and categorized as less than 13 years or 13 years or older. The study country was used to record the country's 2015 HDI ranking (United Nations Development Program [UNDP], 2015). The HDI is a composite of life expectancy at birth, mean and expected years of schooling, and gross national income per capita; countries are ranked from “low” to “very high” (UNDP, 2015). No studies in this meta‐analysis were from countries in the low HDI category. Studies were coded as self‐report if the child completed the outcome measure and parent‐report if the parent completed the outcome measure.

### Data Analysis

We used descriptive statistics to assess the types of studies included in the analysis. Using Comprehensive Meta Analysis (CMA; Version 3) software, we calculated Pearson's *r* effect sizes for each study. For studies in which effect sizes had been calculated using a different method, we used CMA software to convert effect sizes to correlation coefficients. For studies that provided multiple measures within internalizing or externalizing domains or multiple effect sizes based on individual disaster exposure measure items, we calculated averages of the effect sizes (Card, 2012). When a study included three cities with differing levels of exposure, we calculated weighted effect sizes to combine the two cities with the most devastation or exposure (Higgins & Deeks, 2011). Heterogeneity was assessed using a Q statistic, which follows a central chi‐square distribution. When Q was significant, we rejected the null hypothesis, suggesting significant heterogeneity among studies. Categorical moderators using an analysis of variance (ANOVA) approach were used to follow up on heterogeneity within and between groups of studies (Card, 2012). Given the range of age, country, and the outcome measurements used, we used a random‐effects model.

We assessed publication bias using a funnel plot (Light & Pillemer, 1984) to examine the association between the effect size and standard error of each study for each model. The funnel plot was assessed using a sensitivity analysis (Veeva & Woods, 2005). This method has allowed researchers to obtain a more accurate estimate in meta‐analyses with relatively small sample sizes (fewer than 100 studies), compared to more traditional approaches, by using a set of fixed weights to estimate an effect size based on modest and extreme publication bias (Vevea & Woods, 2005).

## Results

### Internalizing Model

We gathered a total of 70 effect sizes from 62 studies that included a total of 376,990 participants (see Table 1 for descriptive statistics) to examine the impact of natural disaster exposure on non‐PTSS internalizing problems. Note that 324,570 participants came from one article that surveyed a database of the population of Sweden and compared tsunami survivors to the nonexposed population (Arnberg et al., 2015). There were 63 outcomes measured in the calculated effect size for the 62 studies (one study used two different outcomes based on age group assessed), including depression only (*n* = 27), a combination of anxiety and depression symptoms (*n* = 20), internalizing composites from either the Child Behavior Checklist (CBCL; Achenbach & Rescorla, 2001) or the Behavior Assessment Scale for Children (BASC; Reynolds & Kamphaus, 2015; *n* = 6), emotion/emotion dysregulation (*n* = 4), somatic symptoms (*n* = 2), internalizing disorder diagnoses (*n* = 2), anxiety (*n* = 1), and fear (*n* = 1).

**Table 1:** Descriptive Statistics of Studies Included in Meta‐Analysis

	Internalizing (*k* = 62)	Externalizing (*k* = 26)
Mean age, years (weighted)^a^	12.61	9.59
Female^b^	26,226	11,529
Male^b^	23,532	15,618
Disaster type^c^		
Blizzard	0	1
Earthquake	24	7
Flood	3	1
Hurricane/typhoon	25	13
Hurricane Katrina	16	8
Tornado	3	2
Tsunami	4	1
Volcanic eruption	1	0
Wildfire	3	1
Publication year		
2010–2017	33	12
2000–2009	24	9
1990–1999	5	4
Before 1990	0	1
Continent of study^c^^,^ d		
Asia	24	8
Europe	7	1
North America	30	17
Oceania	1	0
South America	1	0
Disaster exposure measure		
Established measure	22	9
Other survey^e^	21	8
Geographic group comparison relative to disaster	13	7
Pre/post disaster design	3	2
Single item measure	2	0
National register	1	0

*Note*.^a^Weighted mean age only across studies reporting a mean age. ^b^Gender not available in four internalizing and two externalizing studies. ^c^One internalizing study was conducted in both the United States and South America for two different disaster types, so *k* = 63 for countries and disasters in the internalizing model. ^d^Countries by continent were Armenia, Bangladesh, China, India, Iran, Sri Lanka, Taiwan, Thailand, Turkey (Asia); Greece, Italy, Poland, Sweden (Europe); Cayman Islands, Nicaragua, USA (North America); New Zealand (Oceania); Chile (South America). ^e^“Other survey” indicates disaster exposure was measured using a survey designed for that particular study rather than an established measure.

Table 2 presents a stem and leaf plot of the effect sizes for studies included in the analysis. Significant heterogeneity was found, *Q* = 1434.39, *p* < .001, *df* = 69, with low variability in the studies, τ^2^ = 0.03. The overall average effect size of disasters on non‐PTSS internalizing problems was *r* = .18, 95% CI [0.14, 0.22], *z* = 8.76, *p* < .001. This effect size was small but significant. Removal of the Swedish population‐based study did not change the finding, *r* = .19, *p* < .001. To further assess severity of exposure, we then examined only studies that used a continuous measure of disaster exposure and found the overall effect size remained small but significant, *r* = .17; 95% CI [0.13, 0.20], *z* = 9.35, *p* < .001, *k* = 47. When we performed moderator analyses (Table 3), we found a between‐group difference for HDI categories. Studies in the medium HDI category had a significantly higher mean correlation between disaster exposure and non‐PTSS internalizing problems than those in the high, *Q*_between_ = 37.28, *df* = 1, *p* < .001, and very high categories, *Q*_between_ = 18.89, *df* = 1, *p* < .001. We found no difference between the high and very high HDI groups, *p* = .623. Natural disaster exposure had a similar effect on internalizing problems regardless of age, time since the disaster, or reporter type.

**Table 2:** Stem and Leaf Plot of Effect Sizes

Internalizing Model		Externalizing Model	
Stem	Leaf	Stem	Leaf
−.5		−.5	4
−.4		−.4	7
−.3		−.3	
−.2		−.2	
−.1	2, 4, 9	−.1	2
−.0	2, 6, 7, 9	−.0	6, 8, 8, 8
.0	0, 1, 1, 2, 2, 4, 5, 5, 5, 7, 8, 9, 9	.0	0, 0, 1, 3, 4, 4, 5, 6, 7, 9, 9, 9
.1	0, 1, 1, 1, 2, 2, 3, 3, 4, 4, 4, 4, 5, 5, 5, 6, 6, 6, 7, 7, 7, 7, 7, 8, 9, 9, 9	.1	1, 1, 2, 4, 5, 5, 5, 6, 8, 9
.2	0, 0, 2, 2, 2, 2, 4, 6	.2	
.3	0, 0, 0, 0, 1, 4, 5, 7, 8	.3	
.4	0, 1, 3, 6	.4	
.5		.5	
.6	7	.6	
.7		.7	0
.8	9	.8	1

**Table 3:** Results of Moderator Analysis for Internalizing and Externalizing Symptoms

	Internalizing Model						Externalizing Model					
	*k*	ES	95% CI	*z*	*Q*_between_	*df*	*k*	ES	95% CI	*z*	*Q*_between_	*df*
Average time since disaster												
Less than 1 year	46	0.16	[0.11, 0.21]	5.99***	2.95	1	16	0.03	[−0.06, 0.11]	0.63	3.18	1
One year or more	24	0.23	[0.16, 0.30]	6.50***			15	0.13	[0.05, 0.20]	3.32***		
Average age, years												
≤ 12	33	0.17	[0.10, 0.23]	5.12***	0.48	1	19	0.08	[−0.01, 0.16]	1.78	0.00	1
≥ 13	35	0.20	[0.14, 0.25]	6.49***			11	0.08	[−0.03, 0.18]	1.45		
HDI rank												
Very high	41	0.16	[0.11, 0.21]	6.57***	34.19***	2	21	0.04	[−0.02, 0.10]	1.27	37.36***	2
High	24	0.15	[0.09, 0.20]	4.88***			7	0.04	[−0.02, 0.10]	1.27		
Medium	5	0.56	[0.44, 0.66]	7.99***			3	0.54	[0.40, 0.65]	6.83***		
Reporter												
Parent	16	0.20	[0.11, 0.29]	4.40***	0.20	1	17	0.16	[0.07, 0.24]	3.57***	6.06*	1
Self	51	0.18	[0.13, 0.23]	7.36***			11	−0.01	[−0.12, 0.09]	−0.26		

*Note*. *df* = degree of freedom. HDI = Human Development Index.

*p < .05. **p < .01. ***p < .001.

Given the possible changes in rates of non‐PTSS internalizing problems from childhood to adolescence, we conducted a follow‐up analysis to examine the moderating role of time since disaster by age group. Among the group with an average age of 13 years or higher, the link between disaster exposure and non‐PTSS internalizing problems was significant at both less than 1 year, *r* = .14, 95% CI [0.06, 0.22], *k* = 23, *z* = 3.29, *p* < .001, and greater than 1 year, *r* = .30, 95% CI [0.19, 0.40], *k* = 12, *z* = 5.29, *p* < .001. Further, we found significant between‐group heterogeneity, *Q*_between_ = 5.43, *df* = 1, *p* = .019. This suggests a moderator effect for time since disaster in older youth, such that the effect of disaster exposure on non‐PTSS internalizing problems was stronger among youth who were assessed at least 1 year postdisaster compared to less than 1 year. No between‐group heterogeneity was found for the group with an average age of 12 years or younger, *Q*_between_ = 0.32, *df* = 1, *p* = .572.

When we examined the potential for publication bias, the funnel plot (see Appendix C) demonstrated some asymmetry in the cluster of studies, with more studies clustering to the right of the *y*‐axis relative to the estimated effect size. This indicates that there may be one‐tailed publication bias in our findings (Vevea & Woods, 2005). Moderate and severe one‐tailed publication bias–corrected estimates were calculated based on the interpretation of a one‐tailed bias (Vevea & Woods, 2005). The corrected effect size estimate based on a moderate one‐tailed selection pattern was *r* = .14. A severe one‐tailed selection indicated a significantly reduced effect size estimate of *r* = −.52. Thus, if there was moderate publication bias in our original findings, our estimate would be slightly reduced from our original estimated effect size. If there was severe publication bias, the estimated effect size would be greatly reduced from our original finding and our conclusions would be much different. Based on the broader literature that links trauma exposure and mental health, it is unlikely that such extreme negative findings would be the outcome of exposure to natural disasters on non‐PTSS internalizing problems.

### Externalizing Model

The externalizing model comprised 26 studies (24 of which overlapped with the internalizing model studies) and included a total of 31 effect sizes and 27,496 participants with which to examine the impact of disasters on externalizing problems (see Table 1 for descriptive data). Outcomes assessed included aggression (*n* = 6); externalizing composites from the BASC or CBCL (*n* = 6); other externalizing composites (*n* = 7); externalizing disorder diagnoses (*n* = 3); and behavior problems, such as acting out (*n* = 1), anger (*n* = 1), hostility (*n* = 1), and deviance (*n* = 1).

Table 2 presents a stem and leaf plot of the effect sizes for the studies in the externalizing model. The test for heterogeneity was significant, *Q* = 558.73, *df* = 30, *p* < .001, with low variability in the studies, τ^2^ = 0.02. The overall average effect size of natural disasters on externalizing problems in the random effects model was *r* = .08, 95% CI [.03, .14], *z* = 3.05, *p* = .002. To further assess severity of exposure, we then examined only studies that used a continuous measure of disaster exposure and found a small but significant overall effect size, *r* = .08, 95% CI [.04, .12], *z* = 3.58, *p* < .001*, k* = 20.

The moderator analyses of the externalizing model (Table 3) indicated a significant between‐group difference in reporter, with a stronger association between disaster exposure and externalizing problems among the studies that used parent report of externalizing problems compared to those that used self‐report, *p =* .014. We also found a significant between‐group difference in the country HDI categories. Studies in countries with medium HDI rankings had a significantly higher mean correlation between disaster exposure and externalizing problems than studies in countries with high or very high HDI rankings, *p* < .001. However, findings should be interpreted with caution given the low number of studies conducted in countries with medium HDI rankings. We found no significant difference between the studies conducted in countries with high and very high HDI rankings nor in the analyses of age or time since disaster. We did not conduct further probing of time since disaster within age given the limited number of studies in this model.

When we examined the potential for publication bias, the funnel plot (see Appendix C) did not appear to indicate significant asymmetry on either side of the estimated overall effect size, suggesting publication bias may not be present. Thus, we further examined the potential for publication bias by considering both one‐tailed and two‐tailed publication bias–corrected estimates (Vevea & Woods, 2005). The moderate one‐tailed effect size estimate was *r* = .01 and the severe one‐tailed effect size was *r* = −.52. Both the moderate and severe two‐tailed effect size estimates were *r* = .08. Thus, unless the literature resulted in extreme bias toward only highly positive findings, which the funnel plot did not indicate, it is likely that our findings were not highly influenced by publication bias.

## Discussion

To our knowledge, this study was the first meta‐analysis to examine the impact of exposure to natural disasters on a range of internalizing problems beyond PTSS as well as externalizing problems in youth. Natural disaster exposure had a small but significant effect on non‐PTSS internalizing and externalizing problems. Although small, the effect size obtained for non‐PTSS internalizing problems, *r* = .18, was similar to that obtained in a meta‐analysis of disaster exposure and PTSD in youth (*r* = .16 for the subset of studies looking at natural disasters; Furr et al., 2010). We found substantially more research that met inclusion criteria on non‐PTSS internalizing problems, *k* = 62, than externalizing problems, *k* = 26. The number of studies that examined non‐PTSS internalizing problems in our meta‐analysis is similar to the number that examined only PTSS in the aftermath of natural disaster exposure in a study by Furr et al. (2010). More research on a broad range of MH consequences is needed to promote comprehensive assessment and intervention.

Indeed, our findings suggest that providers should screen for outcomes in addition to PTSS, such as depression, panic, anxiety, and aggressive behavior, when working with youth who have been exposed to natural disasters. Although many trauma‐focused interventions primarily focus on PTSS reduction (e.g., trauma‐focused cognitive behavioral therapy; Cohen, Mannario, & Deblinger, 2006), an appreciation of the range of problems patients may experience can direct treatment selection, sequencing, and adaptation. Developing and/or validating interventions to include reducing additional internalizing and externalizing problems should be a priority for the field. In the absence of such interventions, MH providers should assess a range of problems in disaster‐exposed youth and screen for parental mental illness and parenting stress, which may co‐occur or even precipitate externalizing problems in this population (Scaramella et al., 2008).

We also examined key moderators of the association between natural disaster exposure and non‐PTSS internalizing and externalizing behavior problems in youth. The association between natural disaster exposure and non‐PTSS internalizing and externalizing reactions was similar across age groups. An examination of diverse MH outcomes associated with trauma exposure in both children and adolescents is needed due to potential differences in these outcomes and their association with trauma exposure at younger and older ages (Dunn et al., 2017). We found a stronger association between disaster exposure and externalizing problems for parent report compared to child report. Relatedly, Spell and colleagues (2008) previously identified a link between disaster exposure and child externalizing problems among mothers with high levels of psychological distress. We also found a stronger association between disaster exposure and internalizing and externalizing problems in medium HDI nations compared to high and very high HDI nations. This result was consistent with a prior systematic review by Norris and colleagues (2002) in which the authors found the effects of a disaster were more severe in developing nations with limited financial resources for both individuals and communities compared to the effects of disasters in the United States. Taken together, it is crucial to examine cultural and contextual factors that influence postdisaster outcomes in youth.

We found no significant difference for how soon the study assessments were completed after the disaster in association with either outcome, which highlights the importance of continuity of care both immediately after a disaster and over the long term. One example of an intervention developed to address concerns following the aftermath of disasters is Skills for Psychological Recovery (Berkowitz et al., 2010). Evaluating the effectiveness of this and other assessment and intervention approaches beyond the immediate aftermath of disaster exposure is essential. In our study, there was a stronger association between disaster exposure and non‐PTSS internalizing problems among older youth who were assessed more than 1 year postdisaster compared to those assessed within 1 year, indicating that taking both a developmental and longitudinal approach to assessing and addressing postdisaster psychopathology is important.

This meta‐analysis revealed a growing empirical focus on how natural disasters impact diverse child internalizing and externalizing behavior problems. Next to PTSS, the most common MH issue the available studies addressed was other internalizing reactions. However, the increasing focus on externalizing problems is promising (e.g., Lowe et al., 2013). We found several studies that have pushed the field into newer areas of inquiry, such as exploring culture‐bound syndromes (e.g., Rubens, Felix, Vernberg, & Canino, 2014), sleep problems (e.g., Geng, Fan, Mo, Simandl, & Liu, 2013), posttraumatic growth (Felix et al., 2015), prosocial behavior (Sprague et al., 2015), and sense of community (e.g., Bokszczanin, 2012). Such inquiries should continue, as they reflect the complexity and diversity of postdisaster adjustment. The disaster MH field needs more empirical attention on these more nuanced areas and the ways that factors in children's ecological systems promote postdisaster adaptation.

We were unable to explore some potentially important moderators due to limits of the available literature. To examine gender and race/ethnicity as moderators, a large enough sample of studies would need to provide effect sizes on the association between disaster exposure and non‐PTSS internalizing or externalizing problems by demographic group (sample demographics are not enough). Even in the meta‐analysis on PTSD by Furr and colleagues (2010), only half the included studies provided sufficient information for a gender analysis. Future studies should provide this essential statistical information so that it can be assessed in subsequent meta‐analytic research.

Beyond our statistical findings, our search for relevant articles and common reasons for article exclusion brought to light several notable limitations in the published disaster MH research. Our field needs to define what constitutes “child” versus “adult,” as this operational definition can vary by culture. There were 37 studies that initially met our literature search criteria but were ultimately excluded because outcomes for adolescents and adults were combined. When authors conducted age‐group analyses in studies with large age ranges, their “young adult” category typically spanned from 16 to 24 years of age, combining adolescents with people in their 20s. If sample size allows, researchers should address age‐related influences on adjustment, as previous research has shown important differences in vulnerability to negative outcomes across age groups (Felix et al., 2011; Norris et al., 2002).

The measurement of disaster exposure varied greatly across studies. Unfortunately, several studies were excluded because they did not measure disaster exposure. Instead, these studies reported rates of MH problems in an exposed sample without any comparative information. Among the studies that did measure exposure, the measurements varied and included geographically defined regions of exposure (e.g., high, low, or no impact areas), pre‐ and postdisaster comparisons of MH problems (e.g., prospective studies), individual questions, and a survey or composition (sum) of items. A survey or composition of items is beneficial for measuring the range of potential disaster experiences encountered in the disaster impact and recoil phase and provides a clear statistic to use in a meta‐analysis. The individual‐item approach allows for inclusion of key or marker items, preidentified by the authors as those that could potentially make a significant impact on adjustment (e.g., death of a parent, loss of home), and assesses the influence each item has on adjustment. This is done because sum scores, when items are not weighted, can treat all exposure experiences equally (Netland, 2005) even when it is known that certain experiences (e.g., injury or death) may have a stronger impact than others (e.g., seeing flames). However, this individual‐item approach can make it harder to aggregate information for meta‐analysis. The field needs continued advancement in the measurement of natural disaster exposure and discernment of an approach to weighting key losses when determining an exposure severity scale.

Finally, it is promising that editors of peer‐reviewed journals are increasingly requiring authors to include correlation tables, effect sizes, and other statistical information that will aid other researchers not only in replication but also with meta‐analysis efforts. It was disheartening to exclude studies that measured the constructs of interest but did not provide the needed information to compute an effect size. Sometimes, this was because the purpose of the study was not to explore the association between disaster exposure and MH; therefore, no effect size was calculated on this specific association. Had the authors of those studies included statistics on this association, such as through a correlation table, their studies could have been included. By raising these issues, we hope our field will respond, leading to a clearer, more robust understanding of child disaster MH outcomes.

Results must be interpreted considering the relatively small number of included studies, which may have impacted our ability to identify significant moderators. Also, we only included studies that provided enough data to calculate effect sizes. Publication bias analyses indicated that little difference would have been found if moderate bias was present in both models. In the internalizing model, however, if a severe bias toward studies with positive findings was present, then the outcome would be markedly different. Although we took measures to minimize the likelihood of bias, including requests for unpublished data, inclusion of dissertation data, and contacting authors whose published papers did not include enough information to include in the current analysis, findings should be interpreted in light of the results from the publication bias analysis. Finally, a growing body of research shows that symptoms of internalizing and externalizing problems, including PTSS, may co‐occur in youth exposed to disasters (Adams et al., 2015; Lai et al., 2013). More research is needed to understand the development and sustainment of individual and co‐occurring symptoms following disaster exposure, including an update to the PTSD and depression meta‐analyses previously conducted, as this will be important for intervention development. Most studies assessed symptoms and did not assess whether a participant met diagnostic criteria for a disorder nor did they rule out other disorders with common symptoms. This limitation affected our meta‐analysis by limiting our ability to rule out the possibility that PTSD symptoms are reflected in our findings, just as it affected Furr and colleagues’ (2010) ability to rule out the influence of other internalizing problems on symptoms of PTSD.

In sum, this study is a step forward in exploring the diversity of problems that youth may experience following a natural disaster. Researchers should measure a range of internalizing and externalizing problems as well as other concerns (e.g., substance use) from both a dimensional and diagnostic categorical approach to ensure that they are capturing the full impact of natural disaster exposure on youth. Moving beyond measuring symptoms of distress to understanding how natural disaster exposure and associated long‐term stressors may impact other areas of well‐being (e.g., quality of interpersonal relationships, prosocial behavior, emotion regulation, and health behaviors) is needed. This will help enhance the empirical base so that evidence‐informed, comprehensive strategies can be developed for use in the aftermath of disasters.

## Supporting information

Appendix A. Studies Included in Meta‐Analysis
Appendix B. Descriptive Information of Studies Included in Meta‐Analysis
Appendix C. Funnel plots for a) internalizing model and b) externalizing model
