# Supplementary material for: A Meta‐Analysis of the Impact of Natural Disasters on Internalizing and Externalizing Problems in Youth
Source: J Trauma Stress. 2018 Jun 5;31(3):332–41. doi: 10.1002/jts.22292 (PMC6055700; doi:10.1002/jts.22292)
Supplement: Supplementary file 1 — Appendix A. Studies Included in Meta‐Analysis Appendix B. Descriptive Information of Studies Included in Meta‐Analysis Appendix C. Funnel plots for a) internalizing model and b) externalizing model [file JTS-31-332-s001.docx]

**Appendix A. Studies Included in Meta-Analysis**

Adams, Z. W., Sumner, J. A., Danielson, C. K., McCauley, J. L., Resnick, H. S., Grös, K., ... & Ruggiero, K. J. (2014). Prevalence and predictors of PTSD and depression among adolescent victims of the Spring 2011 tornado outbreak*. Journal of Child Psychology and Psychiatry, 55,* 1047-1055. doi: 10.1111/jcpp.12220

Arnberg, F. K., Gudmundsdóttir, R., Butwicka, A., Fang, F., Lichtenstein, P., Hultman, C. M., & Valdimarsdóttir, U. A. (2015). Psychiatric disorders and suicide attempts in Swedish survivors of the 2004 Southeast Asia tsunami: A 5 year matched cohort study. *The Lancet Psychiatry, 2*, 817-824. doi: 10.1016/S2215-0366(15)00124-8

Banks, D. M., & Weems, C. F. (2014). Family and peer social support and their links to psychological distress among hurricane-exposed minority youth. *American Journal of Orthopsychiatry, 84,* 341-352. doi:10.1037/ort0000006

Belter, R. W., Dunn, S. E., & Jeney, P. (1991). The psychological impact of Hurricane Hugo on children: A needs assessment. *Advances in Behaviour Research and Therapy, 13,* 155-161. doi:10.1016/0146-6402(91)90003-S

Bhushan, B., & Sathya Kumar, J. (2009). Emotional distress and posttraumatic stress in children: the impact of direct versus indirect exposure. *Journal of Loss and Trauma, 14*, 35-45. doi:10.1080/15325020802537047

Bokszczanin, A. (2002). Long-term negative psychological effects of a flood on adolescents. *Polish Psychological Bulletin*, *33*, 55-62.

Burke, J. D., Borus, J. F., Burns, B. J., Millstein, K. H., & Beasley, M. C. (1982). Changes in children's behavior after a natural disaster. *The American Journal of Psychiatry, 139,* 1010-1014. doi: 10.1176/ajp.139.8.1010

Burnham, J. J., Hooper, L. M., Edwards, E. E., Tippey, J. M., McRaney, A. C., Morrison, M. A., ... & Woodroof, E. K. (2008). Examining children's fears in the aftermath of Hurricane Katrina. *Journal of Psychological Trauma, 7,* 253-275. doi: 10.1080/19322880802492229

Chui, C. H., Ran, M. S., Li, R. H., Fan, M., Zhang, Z., Li, Y. H., ... & Fang, D. Z. (2017). Predictive factors of depression symptoms among adolescents in the 18-month follow-up after Wenchuan earthquake in China. *Journal of Mental Health, 26,* 36-42. doi: 10.1080/09638237.2016.1276542

Dogan Ates, A. (2004). An examination of adolescents' post-disaster experiences and reactions following the 1999 Marmara Earthquake (Turkey) (AAI3127020). Available from PsycINFO. (620631520; 2004-99018-263).

Durkin, M. S., Khan, N., Davidson, L. L., Zaman, S. S., & Stein, Z. A. (1993). The effects of a natural disaster on child behavior: evidence for posttraumatic stress. *American Journal of Public Health, 83*, 1549-1553. doi:10.2105/AJPH.83.11.1549

Ekşi, A., Braun, K. L., Ertem-Vehid, H., Peykerli, G., Saydam, R., Toparlak, D., & Alyanak, B. (2007). Risk factors for the development of PTSD and depression among child and adolescent victims following a 7.4 magnitude earthquake. *International Journal of Psychiatry in Clinical Practice, 11,* 190-199. doi:10.1080/13651500601017548

Erkan, S. (2009). The effects of earthquakes on the behavioral and emotional problems of preschoolers. *The Social Sciences, 4*, 347-354.

Fan, F., Zhang, Y., Yang, Y., Mo, L., & Liu, X. (2011). Symptoms of posttraumatic stress disorder, depression, and anxiety among adolescents following the 2008 Wenchuan earthquake in China. *Journal of Traumatic Stress, 24,* 44-53. doi:10.1002/jts.20599

Feitelberg, S. A. (2007). Response to Hurricane Ivan in Grand Cayman: Culture, resilience, and children (AAI3255521). Available from PsycINFO. (622029711; 2007-99018-314).

Felix, E., Hernández, L. A., Bravo, M., Ramirez, R., Cabiya, J., & Canino, G. (2011). Natural disaster and risk of psychiatric disorders in Puerto Rican children. *Journal of Abnormal Child Psychology, 39,* 589-600. doi: 10.1007/s10802-010-9483-1

Felton, J. W., Cole, D. A., & Martin, N. C. (2013). Effects of rumination on child and adolescent depressive reactions to a natural disaster: the 2010 Nashville flood. *Journal of Abnormal Psychology, 122*, 64-73. doi: 10.1037/a0029303

Feo, P., Di Gioia, S., Carloni, E., Vitiello, B., Tozzi, A. E., & Vicari, S. (2014). Prevalence of psychiatric symptoms in children and adolescents one year after the 2009 L'Aquila earthquake. *BMC Psychiatry, 14,* 270-282. doi: 10.1186/s12888-014-0270-3

Giannopoulou, I., Strouthos, M., Smith, P., Dikaiakou, A., Galanopoulou, V., & Yule, W. (2006). Post-traumatic stress reactions of children and adolescents exposed to the Athens 1999 earthquake. *European Psychiatry, 21*, 160-166. doi: 10.1016/j.eurpsy.2005.09.005

Gil‐Rivas, V., & Kilmer, R. P. (2013). Children's adjustment following Hurricane Katrina: The role of primary caregivers. *American Journal of Orthopsychiatry, 83,* 413-421. doi: 10.1111/ajop.12016

Goenjian, A. K., Pynoos, R. S., Steinberg, A. M., Najarian, L. M., Asarnow, J. R., Karayan, I., ... & Fairbanks, L. A. (1995). Psychiatric comorbidity in children after the 1988: earthquake in Armenia. *Journal of the American Academy of Child & Adolescent* *Psychiatry, 34*, 1174-1184. doi:10.1097/00004583-199509000-00015

Goenjian, A. K., Molina, L., Steinberg, A. M., Fairbanks, L. A., Alvarez, M. L., Goenjian, H. A., & Pynoos, R. S. (2001). Posttraumatic stress and depressive reactions among Nicaraguan adolescents after Hurricane Mitch. *American Journal of Psychiatry, 158,* 788-794. doi:10.1176/appi.ajp.158.5.788

Goenjian, A. K., Walling, D., Steinberg, A. M., Roussos, A., Goenjian, H. A., & Pynoos, R. S. (2009). Depression and PTSD symptoms among bereaved adolescents 6½ years after the 1988 Spitak earthquake. *Journal of Affective Disorders, 112,* 81-84. doi: 10.1016/j.jad.2008.04.006

Hardin, S. B., Weinrich, M., Weinrich, S., Hardin, T. L., & Garrison, C. (1994). Psychological distress of adolescents exposed to Hurricane Hugo. *Journal of Traumatic Stress, 7*, 427-440. doi: 10.1002/jts.2490070308

Hensley, L., & Varela, R. E. (2008). PTSD symptoms and somatic complaints following Hurricane Katrina: the roles of trait anxiety and anxiety sensitivity. *Journal of Clinical Child & Adolescent Psychology, 37*, 542-552. doi: 10.1080/15374410802148186

Houlihan, D., Ries, B. J., Polusny, M. A., & Hanson, C. N. (2008). Predictors of behavior and level of life satisfaction of children and adolescents after a major tornado. *Journal of Psychological Trauma, 7*, 21-36. doi: 10.1080/19322880802125902

Huzziff, C. A., & Ronan, K. R. (1999). Prediction of children's coping following a natural disaster—the Mount Ruapehu eruptions: a prospective study. *Australasian Journal of Disaster and Trauma Studies, 1999,* 1-12.

Jia, Z., Tian, W., He, X., Liu, W., Jin, C., & Ding, H. (2010). Mental health and quality of life survey among child survivors of the 2008 Sichuan earthquake. *Quality of Life Research, 19*, 1381-1391. doi: 10.1007/s11136-010-9703-8

Jones, R. T., Ribbe, D. P., Cunningham, P. B., Weddle, J. D., & Langley, A. K. (2002). Psychological impact of fire disaster on children and their parents. *Behavior Modification, 26*,163-186. doi: 10.1177/0145445502026002003

Kadak, M. T., Nasıroğlu, S., Boysan, M., & Aydın, A. (2013). Risk factors predicting posttraumatic stress reactions in adolescents after 2011 Van earthquake. *Comprehensive Psychiatry, 54*, 982-990. doi: 10.1016/j.comppsych.2013.04.003

Kalantari, M., & Vostanis, P. (2010). Behavioural and emotional problems in Iranian children four years after parental death in an earthquake. *International Journal of Social Psychiatry, 56*, 158-167. doi: 10.1177/0020764008101854

Khoury, E. L., Warheit, G. J., Hargrove, M. C., Zimmerman, R. S., Vega, W. A., & Gil, A. G. (1997). The impact of Hurricane Andrew on deviant behavior among a multi‐racial/ethnic sample of adolescents in Dade County, Florida: A longitudinal analysis. *Journal of Traumatic Stress, 10*, 71-91. doi: 10.1002/jts.2490100107

Kiliç, C., Kiliç, E. Z., & Aydin, I. O. (2011). Effect of relocation and parental psychopathology on earthquake survivor-children's mental health. *The Journal of Nervous and Mental Disease, 199*, 335-341. doi: 10.1097/NMD.0b013e3182174ffa

Kolaitis, G., Kotsopoulos, J., Tsiantis, J., Haritaki, S., Rigizou, F., Zacharaki, L., ... & Liakopoulou, M. (2003). Posttraumatic stress reactions among children following the Athens earthquake of September 1999. *European Child & Adolescent Psychiatry, 12*, 273-280. doi: 10.1007/s00787-003-0339-x

Kujawa, A., Hajcak, G., Danzig, A. P., Black, S. R., Bromet, E. J., Carlson, G. A., ... & Klein, D. N. (2016). Neural reactivity to emotional stimuli prospectively predicts the impact of a natural disaster on psychiatric symptoms in children. *Biological Psychiatry, 80*, 381-389. doi: 10.1016/j.biopsych.2015.09.008

Kumar, M., & Fonagy, P. (2013). Differential effects of exposure to social violence and natural disaster on children's mental health. *Journal of Traumatic Stress, 26,* 695-702. doi: 10.1002/jts.21874

La Greca, A. M., Lai, B. S., Joormann, J., Auslander, B. B., & Short, M. A. (2013). Children's risk and resilience following a natural disaster: Genetic vulnerability, posttraumatic stress, and depression. *Journal of Affective Disorders, 151*, 860-867. doi: 10.1016/j.jad.2013.07.024

Lau, J. T., Yeung, N. C., Yu, X. N., Zhang, J., Mak, W. W., & Lui, W. W. (2013). Validation of the Chinese version of the Children's Revised Impact of Event Scale (CRIES) among Chinese adolescents in the aftermath of the Sichuan Earthquake in 2008. *Comprehensive Psychiatry, 54*, 83-90. doi: 10.1016/j.comppsych.2012.06.007

Lewis, K. M., Langley, A. K., & Jones, R. T. (2015). Impact of coping efficacy and acculturation on psychopathology in adolescents following a wildfire. *Journal of Child and Family Studies, 24*, 317-329. doi: 10.1007/s10826-013-9838-7

Liao, T. L., Chen, Y. S., Chen, C. Y., & Chien, L. Y. (2014). Self‐reported Internalizing and Externalizing Behaviours among Junior High School Students at 2 and 4 Years after the 921 Earthquake in Taiwan. *Stress and Health, 30,* 265-271. doi: 10.1002/smi.2506

Liu, M., Wang, L., Shi, Z., Zhang, Z., Zhang, K., & Shen, J. (2011). Mental health problems among children one-year after Sichuan earthquake in China: a follow-up study. *PloS One, 6*, e14706-14714. doi: 10.1371/journal.pone.0014706

Lochman, J.E., Vernberg, E., Powell, N.P., Boxmeyer, C.L., Jarrett M., McDonald K,…Kassing, F. (2017). Pre-post tornado effects on aggressive children's psychological and behavioral adjustment through one- year postdisaster. *Journal of Clinical Child and Adolescent Psychology, 46*, 136-149. doi: 10.1080/15374416.2016.1228460

Lowe, S. R., Godoy, L., Rhodes, J. E., & Carter, A. S. (2013). Predicting mothers' reports of children's mental health three years after Hurricane Katrina. *Journal of Applied Developmental Psychology, 34,* 17-27. doi: 10.1016/j.appdev.2012.09.002

Marsee, M. A. (2008). Reactive aggression and posttraumatic stress in adolescents affected by Hurricane Katrina. *Journal of Clinical Child & Adolescent Psychology, 37*, 519-529. doi: 10.1080/15374410802148152

Navarro, J., Pulido, R., Berger, C., Arteaga, M., Osofsky, H. J., Martinez, M., ... & Hansel, T. C. (2016). Children's disaster experiences and psychological symptoms: An international comparison between the Chilean earthquake and tsunami and Hurricane Katrina. *International Social Work, 59,* 545-558. doi: 10.1177/0020872814537850

Papadatou, D., Giannopoulou, I., Bitsakou, P., Bellali, T., Talias, M. A., & Tselepi, K. (2012). Adolescents' reactions after a wildfire disaster in Greece. *Journal of Traumatic Stress, 25*, 57-63. doi: 10.1002/jts.21656

Pfefferbaum, B., Tucker, P., & Nitiéma, P. (2015, August). Adolescent survivors of Hurricane Katrina: a pilot study of hypothalamic–pituitary–adrenal axis functioning. *Child & Youth Care Forum, 44*, 527-547. doi: 10.1007/s10566-014-9297-3

Pullins, L. G., McCammon, S. L., Lamson, A. S., Wuensch, K. L., & Mega, L. (2005). School-based post-flood screening and evaluation: Findings and challenges in one community. *Stress, Trauma, and Crisis, 8*, 229-249. doi: 10.1080/15434610500406343

Robertson, A. A., Morse, D. T., & Baird-Thomas, C. (2009). Hurricane Katrina's impact on the mental health of adolescent female offenders. *Anxiety, Stress, & Coping, 22,* 433-448. doi: 10.1080/10615800802290634

Roussos, A., Goenjian, A. K., Steinberg, A. M., Sotiropoulou, C., Kakaki, M., Kabakos, C., ... & Manouras, V. (2005). Posttraumatic stress and depressive reactions among children and adolescents after the 1999 earthquake in Ano Liosia, Greece. *American Journal of Psychiatry, 162*, 530-537. doi: 10.1176/appi.ajp.162.3.530

Şahin, N. H., Batıgün, A. D., & Yılmaz, B. (2007). Psychological symptoms of Turkish children and adolescents after the 1999 earthquake: Exposure, gender, location, and time duration. *Journal of Traumatic Stress, 20*, 335-345. doi: 10.1002/jts.20217

Salloum, A., Carter, P., Burch, B., Garfinkel, A., & Overstreet, S. (2011). Impact of exposure to community violence, Hurricane Katrina, and Hurricane Gustav on posttraumatic stress and depressive symptoms among school age children. *Anxiety, Stress, & Coping, 24*, 27-42. doi: 10.1080/10615801003703193

Scaramella, L. V., Sohr-Preston, S. L., Callahan, K. L., & Mirabile, S. P. (2008). A test of the family stress model on toddler-aged children's adjustment among Hurricane Katrina impacted and nonimpacted low-income families. *Journal of Clinical Child & Adolescent Psychology, 37*, 530-541. doi: 10.1080/15374410802148202

Scheeringa, M. S., & Zeanah, C. H. (2008). Reconsideration of harm's way: Onsets and comorbidity patterns of disorders in preschool children and their caregivers following Hurricane Katrina. *Journal of Clinical Child & Adolescent Psychology, 37*, 508-518. doi: 10.1080/15374410802148178

Scott, B. G., Lapré, G. E., Marsee, M. A., & Weems, C. F. (2014). Aggressive behavior and its associations with posttraumatic stress and academic achievement following a natural disaster. *Journal of Clinical Child & Adolescent Psychology, 43*, 43-50. doi: 10.1080/15374416.2013.807733

Spell, A. W., Kelley, M. L., Wang, J., Self-Brown, S., Davidson, K. L., Pellegrin, A., ... & Baumeister, A. (2008). The Moderating Effects of Maternal Psychopathology on Children's Adjustment Post–Hurricane Katrina. *Journal of Clinical Child & Adolescent Psychology, 37*, 553-563. doi: 10.1080/15374410802148210

Tang, T. C., Yen, C. F., Cheng, C. P., Yang, P., Chen, C. S., Yang, R. C., ... & Yu, H. S. (2010). Suicide risk and its correlate in adolescents who experienced typhoon‐induced mudslides: a structural equation model. *Depression and Anxiety, 27,* 1143-1148. doi: 10.1002/da.20748

Terranova, A. M., Morris, A. S., Myers, S., Kithakye, M., & Morris, M. D. (2015). Preschool children's adjustment following a hurricane: Risk and resilience in the face of adversity. *Early Education and Development, 26,* 534-548. doi: 10.1080/10409289.2015.994463

Thienkrua, W., Cardozo, B. L., Chakkraband, M. S., Guadamuz, T. E., Pengjuntr, W., Tantipiwatanaskul, P., ... & Tappero, J. W. (2006). Symptoms of posttraumatic stress disorder and depression among children in tsunami-affected areas in southern Thailand. *Journal of the American Medical Association, 296,* 549-559. doi: 10.1001/jama.296.5.549

Vigil, J. M., Geary, D. C., Granger, D. A., & Flinn, M. V. (2010). Sex differences in salivary cortisol, alpha‐amylase, and psychological functioning following Hurricane Katrina. *Child Development, 81*, 1228-1240. doi: 10.1111/j.1467-8624.2010.01464.x

Wang, W., Fu, W., Wu, J., Ma, X. C., Sun, X. L., Huang, Y., ... & Gao, C. G. (2012). Prevalence of PTSD and depression among junior middle school students in a rural town far from the epicenter of the Wenchuan earthquake in China. *PLoS One, 7,* e41665-41675. doi: 10.1371/journal.pone.0041665

Weems, C. F., Pina, A. A., Costa, N. M., Watts, S. E., Taylor, L. K., & Cannon, M. F. (2007). Predisaster trait anxiety and negative affect predict posttraumatic stress in youths after hurricane Katrina. *Journal of Consulting and Clinical Psychology, 75,* 154-159. doi: 10.1037/0022-006X.75.1.154

Ying, L. H., Wu, X. C., & Chen, C. (2013). Prevalence and predictors of posttraumatic stress disorder and depressive symptoms among child survivors 1 year following the Wenchuan earthquake in China. *European Child and Adolescent Psychiatry, 22*, 567-575. doi: 10.1007/s00787-013-0400-3

Zhang, Y., Kong, F., Wang, L., Chen, H., Gao, X., Tan, X., ... & Liu, Y. (2010). Mental health and coping styles of children and adolescent survivors one year after the 2008 Chinese earthquake. *Children and Youth Services Review, 32*, 1403-1409. doi: 10.1016/j.childyouth.2010.06.009

**Appendix B. Descriptive Information of Studies Included in Meta-Analysis**

| **Study Name** | **Sample**  **Size** | **Boys (Girls)** | **Disaster** | **Exposure Measure** | **Internalizing Outcome / Externalizing Outcome** | **HDI Category** | **Mean**  **Time**  **Since**  **Disaster** | **Age:**  **<13/13+** | **Reporter** |
| --- | --- | --- | --- | --- | --- | --- | --- | --- | --- |
| Adams et al. (2014) | 2000 | 982 (1018) | Spring 2011 Tornado Outbreak, US | Other survey | Depression / N/A | Very High | < 1 year | 13+ | self |
| Arnberg et al. (2015) | 324570 |  | Tsunami | National register | Depression and anxiety / N/A | Very High | ≥ 1 year | < 13 | database |
| Ates (2004) | 695 | 339 (356) | Marmara Earthquake | Geographic group comparison relative to disaster | Depression and anxiety / Behavior problems | High | ≥ 1 year | 13+ | self |
| Banks & Weems (2014) | 1290 | 516 (582) | Hurricane Katrina | Established measure | Depression and anxiety / N/A | Very High | ≥ 1 year | 13+ | self |
| Belter et al. (1991) | 246 |  | Hurricane Hugo | Pre/post design | Depression / Behavior problems | Very High | < 1 year | 13+ | self |
| Bhushan & Sathya Kumar (2009) | 231 | 112 (119) | Tsunami | Geographic group comparison relative to disaster | Depression and anxiety / Acting out | Medium | ≥ 1 year | 13+ | parent |
| Bokszczanin et al. (2002) | 335 | 94 (241) | Flood in Poland | Established measure | Depression / N/A | Very High | ≥ 1 year | 13+ | self |
| Burke et al. (1982) | 64 | 33 (31) | Blizzard | Other survey | N/A / Externalizing behaviors | Very High | < 1 | < 13 | parent |
| Burnham et al. (2008) | 496 | 111(128) | Hurricane Katrina | Geographic group comparison relative to disaster | Fear / N/A | Very High | < 1 year | -- | self |
| Chui et al. (2017) | 548 | 238 (310) | Earthquake in Wenchuan | Other survey | Depression / N/A | High | < 1 year | 13+ | self |
| Durkin et al. (1993) | 162 | 85 (77) | Flood in Bangladesh | Other survey | Shy, withdrawn / Aggression | Medium | < 1 year | 13+ | parent |
| Eksi et al. (2007) | 160 | 58 (102) | Earthquake in Turkey (1999) | Other survey | Depression / N/A | High | < 1 year | 13+ | self |
| Erkan et al. (2009) | 482 | 231 (251) | Earthquake in Turkey | Other survey | Internalizing composite / Externalizing composite | High | < 1 year | < 13 | parent |
| Fan et al. (2011) | 2081 | 955 (1126) | Earthquake in Wenchuan | Established measure | Depression and anxiety / N/A | High | < 1 year | 13+ | self |
| Feitelberg (2007) | 129 | 61 (68) | Hurricane Ivan | Established measure | Depression / N/A | Very High | ≥ 1 year | -- | self |
| Felix et al. (2011) | 1886 | 964 (922) | Hurricane Georges | Established measure | Internalizing diagnosis / Externalizing diagnosis | Very High | ≥ 1 year | < 13 | parent and child |
| Felton et al. (2013) | 239 | 106 (133) | Flood in Tennessee | Established measure | Depression / N/A | Very High | < 1 year | < 13 | self |
| Feo et al. (2014) | 1839 | 902 (929) | Earthquake in L'Aquila, Italy | Geographic group comparison relative to disaster | Internalizing composite / Externalizing composite | Very High | ≥ 1 year | < 13 | parent |
| Giannopoulou et al. (2006) | 2037 | 990 (1046) | Earthquake in Athens | Geographic group comparison relative to disaster | Depression / N/A | Very High | < 1 year | 13+ | self |
| Gil-Rivas & Kumar (2013) | 68 | 30 (38) | Hurricane Katrina | Established measure | Depression / N/A | Very High | ≥ 1 year | < 13 | self |
| Goenjian et al. (1995) | 218 | 82 (136) | Earthquake in Armenia | Geographic group comparison relative to disaster | Depression / N/A | High | ≥ 1 year | 13+ | self |
| Goenjian et al. (2001) | 158 | 81 (77) | Hurricane Mitch | Geographic group comparison relative to disaster | Depression / N/A | Medium | < 1 year | 13+ | self |
| Goenjian et al. (2009) | 92 | 34 (58) | Earthquake in Spitak | Geographic group comparison relative to disaster | Depression / N/A | High | ≥ 1 year | 13+ | self |
| Hardin et al. (1994) | 1482 | 726 (756) | Hurricane Hugo | Other survey | Depression and anxiety / Anger | Very High | ≥ 1 year | 13+ | self |
| Hensley & Varela (2008) | 302 | 118 (184) | Hurricane Katrina | Established measure | Somatic symptoms / N/A | Very High | < 1 year | < 13 | self |
| Houlihan et al. (2008) | 95 |  | Tornado in Minnesota | Established measure | Internalizing composite / Externalizing composite | Very High | < 1 year | < 13 | parent |
| Huzziff & Ronan (1999) | 187 | 89 (95) | Mount Ruapehu Eruption | Single item measure | Depression and anxiety / N/A | Very High | < 1 year | < 13 | self |
| Jia et al. (2010) | 596 | 227 (369) | Earthquake in Sichuan (2008) | Established measure | Depression / N/A | High | ≥ 1 year | < 13 | self |
| Jones et al. (2002) | 22 | 8 (14) | Wildfire in Southern CA | Established measure | Internalizing disorder diagnosis / Externalizing disorder diagnosis | Very High | < 1 year | < 13 | parent and child |
| Kadak et al. (2013) | 759 | 417 (342) | Van Earthquake | Other survey | Depression and anxiety / N/A | High | < 1 year | 13+ | self |
| Kalantari & Vostanis (2010) | 166 | 83 (83) | Earthquake in Iran | Geographic group comparison relative to disaster | Emotion, emotion dysregulation / Behavior problems | High | ≥ 1 year | < 13 | parent |
| Khoury et al. (1997) | 4978 | 4488 (490) | Hurricane Andrew | Other survey | N/A / Deviance | Very High | < 1 year | -- | self |
| Kilic et al. (2011) | 104 | 43 (61) | Earthquake | Single item measure | Depression / N/A | High | ≥ 1 year | < 13 | self |
| Kolaitis et al. (2003) | 163 | 99 (116) | Earthquake in Athens | Other survey | Depression and anxiety / N/A | Very High | < 1 year | < 13 | self |
| Kujawa et al. (2016) | 260 | 141 (119) | Hurricane Sandy | Established measure | Internalizing composite / Externalizing composite | Very High | < 1 year | < 13 | parent |
| Kumar et al. (2013) | 196 | 98 (98) | Earthquake in Guharat, India | Geographic group comparison relative to disaster | Emotion, emotion dysregulation / Behavior problems | Medium | ≥ 1 year | < 13 | self |
| La Greca et al. (2013) | 116 | 53 (63) | Hurricane Ike | Established measure | Depression / N/A | Very High | < 1 year | < 13 | self |
| Lau et al. (2013) | 3160 | 1694 (1466) | Earthquake in Sichuan (2008) | Other survey | Depression and anxiety / N/A | High | < 1 year | 13+ | self |
| Lewis et al. (2013) | 185 | 74 (111) | Wildfires in Florida | Established measure | Depression and anxiety / N/A | Very High | < 1 year | 13+ | self |
| Liao et al. (2014) | 12111 | 6093 (6018) | Earthquake in Taiwan | Geographic group comparison relative to disaster | Depression and anxiety / Behavior problems | High | ≥ 1 year | 13+ | self |
| Liu et al. (2011) | 330 | 165 (165) | Earthquake in Sichuan (2008) | Other survey | Depression and anxiety / N/A | High | < 1 year | < 13 | self |
| Lochman et al., 2017) | 360 | 234 (126) | Tornadoes in Alabama | Established measure | Anxiety / Aggression | Very High | < 1 year | < 13 | parent |
| Lowe et al. (2013) | 251 | 118 (133) | Hurricane Katrina | Other survey | Depression and anxiety / Behavior problems | Very High | ≥ 1 year | < 13 | parent |
| Marsee (2008) | 166 | 65 (101) | Hurricane Katrina | Established measure | Emotion, emotion dysregulation / Aggression | Very High | ≥ 1 year | 13+ | self |
| Navarro et al. (2014) | 827 |  | Hurricane Katrina and Chile Earthquake | Established measure | Depression / N/A | Very High | < 1 year | 13+ | self |
| Papadatou et al. (2012) | 1468 | 755 (713) | Wildfire | Established measure | Depression / N/A | Very High | < 1 year | 13+ | self |
| Pfefferbaum et al. (2015) | 23 | 11 (12) | Hurricane Katrina | Geographic group comparison relative to disaster | Depression / N/A | Very High | ≥ 1 year | 13+ | self |
| Pullins et al. (2005) | 612 | 257 (355) | Hurricane Floyd | Other survey | Depression / N/A | Very High | < 1 year | < 13 | self |
| Robertson et al. (2009) | 258 | 0 (258) | Hurricane Katrina | Other survey | Depression and anxiety / N/A | Very High | < 1 year | 13+ | self |
| Roussos et al. (2005) | 1937 | 109 (1090) | Earthquake in Greece | Established measure | Depression / N/A | Very High | < 1 year | 13+ | self |
| Sahin et al. (2007; adolescent | 948 | 462 (486) | Earthquake in Turkey | Other survey | Depression and anxiety / Hostility | High | < 1 year | < 13 | parent |
| Sahin et al. (2007; child) | 1260 | 642 (617) | Earthquake in Turkey | Other survey | Somatization / N/A | High | < 1 year | 13+ | self |
| Salloum et al. (2011) | 122 | 70 (52) | Hurricane Katrina | Other survey | Depression / N/A | Very High | < 1 year | < 13 | self |
| Scaramella et al. (2008) | 102 | 41 (61) | Hurricane Katrina | Pre/post design | Internalizing composite / Externalizing composite | Very High | ≥ 1 year | < 13 | parent |
| Scheeringa & Zeenah (2008) | 70 | 40 (30) | Hurricane Katrina | Other survey | Depression and anxiety / Externalizing diagnosis | Very High | < 1 year | < 13 | parent |
| Scott et al. (2014) | 191 | 105 (86) | Hurricane Katrina | Established measure | N/A / Aggression | Very High | ≥ 1 year | < 13 | self |
| Spell et al. (2008) | 260 | 112 (148) | Hurricane Katrina | Established measure | Internalizing composite / Externalizing composite | Very High | < 1 year | < 13 | parent |
| Tanaka et al. (2016) | 2641 | 1182 (1459) | Earthquake in Wenchuan | Other survey | Depression and anxiety / N/A | High | ≥ 1 year | 13+ | self |
| Tang et al. (2010) | 271 | 124 (147) | Typhoon Morakot | Other survey | Depression / N/A | High | < 1 year | 13+ | self |
| Terranova et al. (2015) | 118 | 67 (51) | Hurricane Katrina | Established measure | Emotion, emotion dysregulation / Aggression | Very High | ≥ 1 year | < 13 | parent |
| Thienkrua et al. (2006) | 371 | 170 (201) | Tsunami | Established measure | Depression / N/A | High | < 1 year | < 13 | self |
| Vigil et al. (2010) | 115 | 71 (44) | Hurricane Katrina | Geographic group comparison relative to disaster | Depression / Aggression | Very High | < 1 year | 13+ | self |
| Wang et al. (2012) | 1841 | 897 (944) | Earthquake in Wenchuan | Other survey | Depression / N/A | High | < 1 year | 13+ | self |
| Weems et al. (2007) | 52 | 30 (22) | Hurricane Katrina | Pre/post design | Depression and anxiety / N/A | Very High | < 1 year | < 13 | self |
| Ying et al. (2015) | 2298 | 1127 (1164) | Earthquake in Wenchuan | Other survey | Depression / N/A | High | < 1 year | < 13 | self |
| Zhang et al. (2010) | 423 | 188 (235) | Earthquake in Sichuan (2008) | Other survey | Depression / N/A | High | < 1 year | 13+ | self |

*Note*. Data in Banks & Weems (2014) within the internalizing model and data in Scott et al. (2014) within the externalizing model come from the same sample. Gender not available in four internalizing and two externalizing studies. “Other survey” indicates disaster exposure was measured using a survey designed for that particular study rather than an established measure. Outcomes listed as “composite” come from either the Child behavior Checklist or the Behavior Assessment Scale for Children. Other combinations within the outcomes are from measures of various internalizing/externalizing outcomes.

**Appendix C. Funnel plots for a) internalizing model and b) externalizing model**

A.

B.
